# Improvements in biomaterial matrices for neural precursor cell transplantation

**DOI:** 10.1186/2052-8426-2-19

**Published:** 2014-07-01

**Authors:** Nolan B Skop, Frances Calderon, Cheul H Cho, Chirag D Gandhi, Steven W Levison

**Affiliations:** Department of Neurology & Neurosciences, Rutgers University-New Jersey Medical School, NJMS-Cancer Center, H-1226, 205 South Orange Ave., Newark, NJ 07103 USA; Department of Neurological Surgery, Rutgers University-New Jersey Medical School, New Jersey Medical School, Newark, NJ 07103 USA; Department of Biomedical Engineering, New Jersey Institute of Technology, Newark, NJ 07102 USA

**Keywords:** Tissue engineering, Neural stem cells, Scaffold, Biomaterials, CNS, Brain injury, TBI, Stroke, Transplantation, Review

## Abstract

Progress is being made in developing neuroprotective strategies for traumatic brain injuries; however, there will never be a therapy that will fully preserve neurons that are injured from moderate to severe head injuries. Therefore, to restore neurological function, regenerative strategies will be required. Given the limited regenerative capacity of the resident neural precursors of the CNS, many investigators have evaluated the regenerative potential of transplanted precursors. Unfortunately, these precursors do not thrive when engrafted without a biomaterial scaffold. In this article we review the types of natural and synthetic materials that are being used in brain tissue engineering applications for traumatic brain injury and stroke. We also analyze modifications of the scaffolds including immobilizing drugs, growth factors and extracellular matrix molecules to improve CNS regeneration and functional recovery. We conclude with a discussion of some of the challenges that remain to be solved towards repairing and regenerating the brain.

## Introduction

Tissue engineering (TE) is a relatively new and expanding field. Using principles from material engineering and molecular biology, tissue engineers develop organic substitutes to support or replace portions of malfunctioning tissues or organs [1]. These substitutes are commonly created using living cells, biomaterial scaffolding and signaling molecules [2]. Studies have shown that cell replacement and tissue repair is improved when engrafted cells are delivered on a biomaterial scaffold. This is because a scaffold can provide structural support for the cells as well as carry necessary factors that enhance their survival and function. TE has been investigated in a variety of organs and is an area of active investigation for central nervous system (CNS) repair. TE for brain injury has not been as widely explored — likely due to the complexity of central neural circuitry. TE has recently gained in popularity with the realization that transplanting stem cells with a biomaterial substrate is more effective. In this article we review progress to date employing TE to promote cell replacement using neural precursors (NPs) to restore neurological function after traumatic brain injuries and stroke. The therapeutic value in transplanting neural precursors is extremely high due to the inability of neurons to undergo mitosis and the incapacity of the brain to repair large injuries on its own.

### Brain injuries

Approximately 1.7 million Americans sustain traumatic brain injuries (TBI) each year as a result of falls, motor vehicle accidents, being struck by objects or assaults. An additional 800,000 individuals are affected by stroke, of which 80% are ischemic and are of varying severity. These numbers do not include major brain injuries caused by infections, tumors or other CNS diseases that account for another large population. Brain injuries are generally classified as mild, moderate or severe depending on the damage sustained. The majority of TBIs are mild, resulting in a change in mental status or state of consciousness. Severe brain injuries may cause amnesia, long periods of unconsciousness, irreversible changes in cognitive (attention and memory), motor (coordination, balance, and limb weakness/paralysis) and sensorimotor function (vision, hearing, and touch), alteration in emotions (anxiety, depression, and personality changes) and sometimes death [3, 4].

### Pathophysiology

Individuals who do not die within the first few months after sustaining a severe brain injury are often left with disabilities and a poor prognosis for the duration of their lives. The acute affects can be observed within the first hours after injury and can be amplified within the first several weeks, generally attributed to the pro-inflammatory response to the injury that can last for months or years [5]. Neuronal damage and cell loss have been extensively documented and characterized in the cerebral cortex, the hippocampus and the thalamus in the acute phase following experimental brain injury [6–9]. The primary damage created by mechanical forces at the moment of the impact is irreversible. In response, immune cells are recruited to the damaged site, whereupon they release cytokines and chemokines triggering a neuroinflammatory reaction that produces a wave of secondary cell death. After a delay, the astrocytes surrounding the injury begin to produce a glial scar. Once formed, this scar tissue creates an inhibitory environment eliminating the possibilities of axonal regeneration due to the formation of a complex extracellular matrix (ECM) [10–12]. This prolonged and progressive pathologic cascade becomes the basis for the deficits in cognitive and motor function that begin in the first hours after TBI and may continue for years.

### Treatment

After a person sustains an injury, the medical team will provide resuscitation procedures, and stabilize vital functions to minimize secondary damage to the brain. Mechanical ventilation is used to support respiration and to maintain lower intracranial pressure. Sensory devices may be surgically placed into the brain cavity to monitor or control intracranial pressure. Surgery may be required to repair hemorrhaged arteries or to eliminate blood clots. Blood, fluid and bone particles can be removed while damaged tissue, blood vessels or the skull can be surgically remodeled in severe cases where there is extensive swelling. Patients are also kept sedated with medications to prevent them from causing any additional injury and to prevent seizures and spasticity. Doctors try to maximize cerebral perfusion pressure and blood flow (which includes oxygen and nutrients being supplied to the brain) while minimizing the swelling caused by pressure that may damage more cells [13, 14]. Pharmaceutical agents also may be used to limit secondary damage to the brain which include: diuretics to reduce edema thus decreasing pressure; anti-seizure drugs to avoid additional brain damage; and coma-inducing drugs because a comatosed brain requires less oxygen to function [15]. Other medications such as analgesics, anti-anxiety agents, anti-depressants, anti-psychotics, muscle relaxants, sedatives and stimulants are also commonly utilized in patients sustaining TBI [16]. To date, however, there are no therapies capable of replacing the neurons lost to brain injuries, thus making full functional recovery after severe TBI impossible.

### Endogenous stem cells

The brain is arguably the most difficult organ to repair after an injury due to the complexity of network of cells that comprise the central nervous system. Its capacity to regenerate is complicated by the inability of neurons to undergo mitosis. Scientists have tried to expand the endogenous stem cells found inside the brain to repair damage after CNS injury. Despite significant work, several problems still exist with this approach. First, few neurons are generated in response to injury, as the vast majority of the new cells that are produced become glia. While infants have significantly larger numbers of neural stem cells (NSCs) than adults, and thus greater potential for repair [17, 18], the NSCs of the immature brain simply do not produce many new neurons after TBI (Goodus et al., Submitted). Another barrier to regeneration from the endogenous stem cells of the brain is that the pools of NSCs are depleted with age [19].

These observations hold for several different CNS injury models. Arvidsson et al. [20] researched the mechanisms of neuronal repair after stroke in an adult rat model and reported that less than 1% of the destroyed neurons are replaced from the endogenous neural precursors (NPs) of the subventricular zone (SVZ). Similar results were obtained in rat models of stroke in the immature animal where cell counts of immature neurons vs. mature neurons revealed that greater than 75% of the newly produced neurons failed to survive. Moreover, of those neurons that did survive, they were predominantly GABAergic interneurons [21, 22]. In a mouse model of TBI the same pattern was seen [23]. Salman et al. [23] found that SVZ cells proximal to the injured area produced a very small percentage of new neurons (not quantified), while the majority became astrocytes. Whether the newly generated neurons died at the injury site or failed to migrate from the SVZ to the damaged region is unclear. However, it is known that in the adult brain, neural progenitors have a difficult time migrating to the injured cortex due to dense white matter tracts [24].

Researchers are looking at pharmacological means to generate a more robust response from SVZ precursors, to stimulate their proliferation, increase their migration to the affected sites and to increase their production of region-appropriate neurons. Major advancements have yet to be made; therefore, alternative solutions have been sought by a number of groups.

### Transplanting exogenous stem cells

Given the limitations of the endogenous NSCs, transplanting exogenous NPs into the injured brain has gained traction as a more appropriate solution to promote CNS regeneration. Yet this raises the issue of which cell type to transplant. Since brain injuries result in the demise of a range of different neuronal cell types as well as the astrocytes and oligodendrocytes that support them, the ideal cell would be one that has the capacity to produce a large repertoire of different neurons and glia. A benefit of transplanting a stem cell is that a stem cell can produce a large number of progeny that are capable of integrating into many regions as neurons and glia to replace missing or dysfunctional neural cells [25–28]. To date, several types of CNS progenitors as well as several neural stem cell lines have been transplanted into the injured brain. Cells possessing the properties of neurons have been observed, however, it is important to note that a significant proportion, regardless of the starting precursor, express the biochemical and phenotypic characteristics of glia [29–38]. Both progenitor and stem cell grafts have been shown to improve functional outcome following TBI [39–42] and in experimental stroke [43–49]. Generally speaking, studies that transplanted progenitors or more differentiated cells have been less successful than studies using neural stem cells in replacing or rebuilding a neural circuit. Although there is no study directly comparing neuron, progenitor and stem cell transplantations, the vast majority of research on CNS regeneration focuses on the use of stem cell or early progenitor therapies. Lineage progression from a stem cell to a mature neuron is a process in which proliferation, migration and multipotential capacity decreases. Bliss et al. [50], transplanted human post-mitotic neurons (from hNT cell line derived from human teratocarcinoma) into a rat model of stroke and noticed low donor cell survival [51]. Although they saw neurite extension from hNT neurons, there was no migration. Conversely, transplanted stem cells have shown excellent migration to lesions even when transplanted into the contralateral hemisphere [51–53]. Poor cell survival in the cell preparation and during the transplantation process has been noted, especially when transplanting more committed cells into the unwelcoming milieu of a focal neocortical injury. Thus stem cell transplantation studies are more commonly observed in CNS therapeutics, whereas neurons and more differentiated cell types are generally avoided. Bone marrow stromal cells have been shown to improve outcome after brain injury and stroke [54–63], but the evidence suggests that the functional improvements obtained are not a result of cell replacement but are due to secreted factors that are neuroprotective.

Immortalized multipotent stem cell lines, such as C17.2 (immortalized mouse neural progenitor cell line), HiB5 (rat hippocampal stem cell line) and MHP-36 (murine neural stem cell line), which display properties of stem cells, have been successfully transplanted into rodents subjected to either ischemic or contusive injuries [64–66]. Upon directly transplanting these cell lines each engrafted and migrated preferentially to areas with active neurodegeneration [67–70]. HiB5 is an immortalized cell line from embryonic day 16 rat hippocampus. Philips et al. [71] transplanted HiB5 cells into the brain one week following fluid percussion brain injury and reported that they reduced cell death in the CA3 region of the hippocampus. MHP-36 is an immortalized mouse hippocampal neuroepithelial stem cell line derived from embryonic day 14 heterozygous H-2Kb-tsA58 transgenic mice (a mouse line containing an inducible Class I antigen transgene). When grafted into rat brains 2–3 weeks after global ischemia or excitotoxic hippocampal injury, MHP-36 cells migrated, improved motor and sensory function and repopulated the lesioned CA1 hippocampal region [72–75]. Sinden et al. and Riess et al. transplanted MHP-36 cells after hypoxia/ischemia and fluid percussion injury respectively [41]. Sinden observed a reduction in lesion size after transplantation while Riess noticed a decrease in learning deficits that correlated with the migration of MHP-36 cells to the injury site [76]. Despite these encouraging results, the MHP-36 cells cause a profound inflammatory response [74].

Studies have also shown that these cell lines, including the immortalized C-17.2 cell, can produce tumors when transplanted following TBI [77]; thus, the likelihood that immortalized stem cell lines might ever be used to treat patients is low. Therefore, many researchers have evaluated grafting primary fetal and early postnatal neural stem cells into the adult CNS to repair the brain after injury. The rationale for using primary cells is that in the future it may be possible to derive similar donor cells from human embryonic stem cells (ESC) or induced pluripotent stem cells (iPSC). Transplanting primary NPs has shown promise as many groups have demonstrated the ability to achieve engraftment and differentiation. Hoane et al. [78] and Gao et al. [79] have reported differentiation of rodent NPs into neurons, and these cells survived one year post-transplantation into the brain. Even though a significant improvement in cognitive recovery was not evident, the researchers did observe greater sensorimotor recovery in these rodents. Calcagnotto et al. [80], transplanted cells from the mouse medial ganglionic eminences (MGE), and demonstrated that these NPs migrated and differentiated into cortical GABAergic interneurons. Local pyramidal neurons were patched and electrophysiological recordings indicated functional integration of the transplanted MGE cells into the host tissue.

These experimental studies suggest that many obstacles have been overcome in the grand quest to heal TBI with exogenous cell transplants, but the extent of neuronal cell replacement has still been variable and few of transplanted cells are retained [81, 82]. Most of the transplanted cells either do not survive [83–85] or differentiate into glial cells instead of neurons [52, 86–88]. This is a concern that the stem cells transplanted do not differentiate into reactive astrocytes that can contribute to glial scarring. Shear et al. [87] and Boockvar et al. [52], found that NG2 positive glial cells were produced upon transplanting NPs and Sun et al. [88], observed that the majority of the precursors that they transplanted became Olig2 positive cells (presumably glia). Ma et al. [86], transplanted NPs (comprised of 4% NSCs) and reported that only 11% of the differentiated cells expressed a neuronal marker. We have generated data that parallel these findings, once again obtaining poor survival of NPs when transplanted directly into the parenchyma following TBI [89]. A new technique or paradigm for overcoming these problems associated with NP transplantation is necessary.

### Brain tissue engineering

A focal traumatic brain injury results in a large number of dead cells and debris that are localized near the region of impact. Macrophages clear away the remnants of dead or dying cells, but the injury creates a harsh, non-permissive environment that lacks nutrients, survival factors and most importantly, a habitable substrate and the extracellular matrix (ECM) that they once resided within [14, 39, 40, 90]. This ECM is a scaffold that provides cells with structural and functional support. It is comprised of interconnected proteins and proteoglycans that create a framework that cells adhere to. Attachment to the individual components of this matrix transduces mechanical signals that regulate both basic and complex cellular processes. The proteins and proteoglycans that comprise the ECM bind to a number of surface receptors found on cells that can affect proliferation, migration, differentiation, survival and other functions [91–94]. Although reactive astrocytes produce ECM molecules in the process of generating the glial scar, this ECM is distinctly different from the normal ECM in the brain functionally, chemically and mechanically [95].

**Figure 1:**
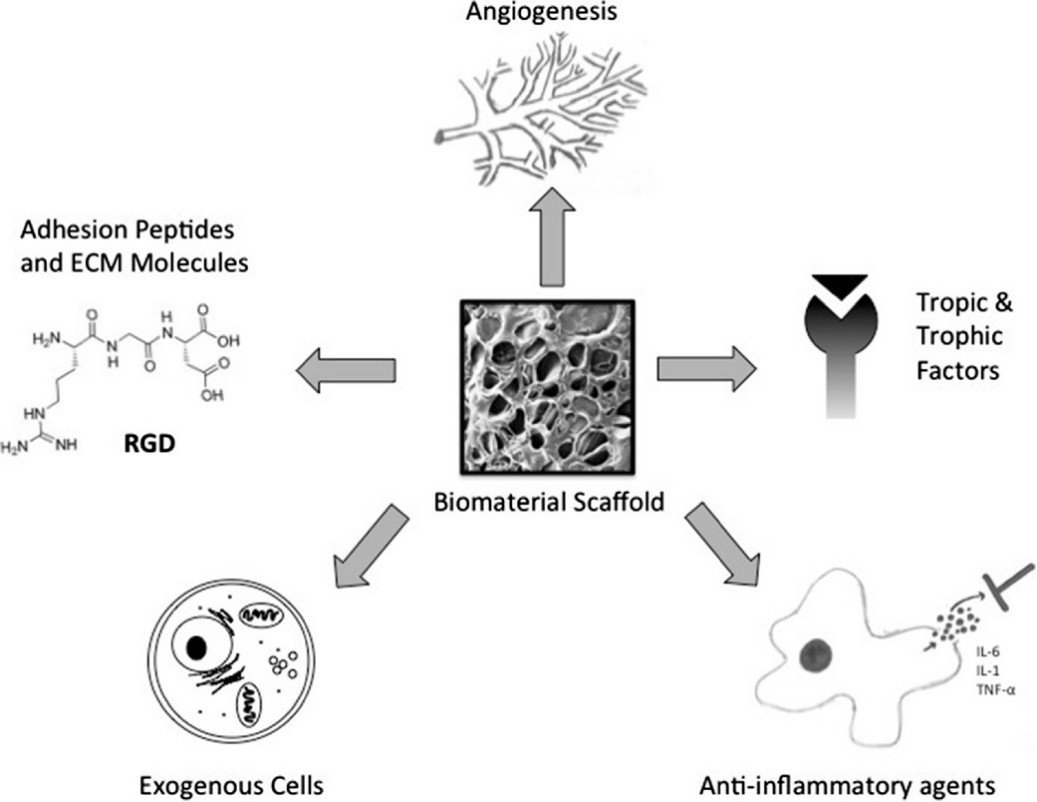
**Modifications made to brain tissue engineered scaffolds to promote tissue repair.** Biomaterial matrices can be designed to incorporate cells (e.g. stem cells and progenitors), trophic and tropic factors to support exogenous or endogenous cells, factors that induce angiogenesis, anti-inflammatory agents and synthetic adhesion molecules or extracellular matrix (ECM) derivatives.

Scaffolds are three-dimensional artificial structures that are created to recapitulate the *in vivo* milieu providing cells with an appropriate microenvironment. Since brain injuries vary in shape and size, scaffolds that form after injection into the wound cavity allow for a one-size-fits-all solution. Several factors must be considered when creating a scaffold for a particular biological application. In the pursuit of the ideal biomaterial design a wish list of desirable functions can be created. The wish list should include:

- ● The scaffold must be nontoxic and biocompatible with transplantable cells. This means that the scaffold should not adversely affect cell function or cell survival.
- ● The scaffold must be biocompatible with the brain tissue environment. The scaffold should not elicit a damaging immune response, be toxic, carcinogenic, or adversely affect the survival of the host cells. Upon degradation the scaffold should not generate toxic, corrosive or acidic byproducts.
- ● The scaffold can maintain the “stemness” of the transplanted cells. Indeed, because stem cells require fewer survival factors than committed cells [96], a transplanted stem cell, as opposed to a committed cell or a differentiated precursor, might stand a better chance of surviving transplantation. Stemness can be maintained by including ligands that will promote self-renewal and proliferation while simultaneously decreasing differentiation.
- ● Biodegradation of the scaffold should be controlled. This rate should be designed to mirror the proliferation and growth of the transplanted cells. In essence, as cells begin to fill the void created by an injury, the implanted support structure should degrade in such a fashion that it remains available while the engrafted cells continue to grow. Importantly, were the scaffold to remain intact it could increase in intracranial pressure, inflammation and fibrous encapsulation with adverse effects.
- ● The scaffold should be injectable. Furthermore, brain injuries evolve and produce lesions of different shapes and sizes. An injectable matrix will accommodate the various types of brain defects that need to be repaired. Instead of designing biomaterial implants with varying shapes and sizes, only one type of injection material would be needed (with varying volume). Economically speaking, this would drastically reduce manufacturing and patient costs.
- ● The scaffold must remain local. It is important that the injected material, whether intact or degraded, does not redistribute into unintended regions of the brain or body. The scaffold should sit in, or around, the lesion cavity until the wound has healed. Diffusion of the scaffold itself, or the transplanted cells into improper locations could have adverse affects.
- ● The scaffold should be porous. Interconnected pores, in the scaffold would promote blood vessel ingrowth, nutrient flow and cellular infiltration. Without these properties the transplanted cells may not survive or integrate into the native tissue due to the lack of nutrients and waste removal.An illustration of modifications and additions made to biomaterial scaffolds for brain TE can be seen in Figure 1.

### Biomaterial scaffold structures

From this list we may narrow down the types of scaffolds and the compositions of biomaterials optimal for use. Since an injectable scaffold is desired, this significantly limits the biomaterials available. Two common designs that would apply would be hydrogel systems and micro- or nano-particle systems. Hydrogels are liquid, but undergo gelation upon injection into the brain. Often times this is achieved through the change in temperature from ambient air temperature of ~ 21°C to the body temperature of ~ 37°C. Alternatively, micro- or nano-particles could be produced varying in configuration from microscopic spheres, irregular particles or as fibers that are subsequently suspended in a liquid or gel for transplantation.

#### Hydrogels

Hydrogels are water-soluble polymer chain networks. They can absorb up to 99 percent water, which makes them a strong candidate for brain scaffolding. They have excellent nutrient and oxygen permeability, allowing cell survival in the scaffold [97]. Hydrogels can also be modified with proteins, glycosaminoglycans (GAGs), cytokines, drugs and other factors that will stimulate cell adhesion and/or growth [98]. Cells are readily encapsulated into hydrogels to replace missing autologous cells. Most importantly, hydrogels form in situ. As their name suggests, they gel following injection into tissues [99]. Furthermore, hydrogels possess elastic properties that are similar to those of natural brain tissue. Hydrogels can be created with low compressive moduli that tend to direct stem cell differentiation toward neural lineages [100–107].

A downside to hydrogels is that cellular migration and outgrowth is often poor due to its weak mechanical structure. In the CNS migration is essential for the initial formation of cortical architectural, for axonal growth and synaptogenesis and for white matter colonization by oligodendrocyte progenitors prior to myelination. Moreover, cells, and in particular neurons, do not extend their neurites through three-dimensional matrices efficiently [102, 108–112]. Neurite outgrowth is best observed on 2-D rigid structures. This is due, in part, because neuronal growth cones require stiff substrates to pull on in order to grow or stretch. The filopodia of many cells have similar properties. Cells placed onto softer substrates are often round and maintain very short processes. Thus a hydrogel will not likely create a suitable environment for radial glial cells (RGCs) that naturally extend their processes long distances to the pial surface of the brain during embryonic development. Another disadvantage in using hydrogels is that their biodegradation is hard to control [113, 114]. Because the majority of hydrogel systems focus on gelation and cytocompatibility, degradation rates are often sacrificed or difficult to manipulate.

#### *Microspheres and microparticle*s

Microspheres and microparticles on the other hand, possess a rigid surface structure, as opposed to the soft structure of hydrogels. Due to their rigidity, the tension that neuronal growth cones require can be created and maintained more easily on microspheres than on hydrogels. Furthermore, microspheres can be transplanted by syringe, whereupon they can mold to the injury dimensions. In addition, microspheres can be fabricated to encapsulate, immobilize and deliver specific growth or trophic factors to aid engraftment and survival of the transplanted cells [115]. A downside in using microspheres is that they may be more difficult to inject than hydrogels, since hydrogels are liquid within the injection syringe and gel upon contact with the brain (usually due to temperature differences) whereas, microparticles typically need to be suspended in an additional solution. Another limitation is the weak elasticity of microparticles. Stiffness might increase neurite outgrowth, although it might also decrease differentiation. Studies have shown that materials constructed with elastic properties similar to that of natural brain tissue are more likely to favor neuronal differentiation [100, 116]. Microspheres are inferior in this regard.

### Natural versus synthetic polymers

The material composition to be used for biomatrix construction must be carefully selected. Biomaterial matrices can be formed using natural or synthetic polymers, each possessing unique characteristics and gelling properties depending on fabrication. Natural polymers have many attractive features for TE. Since these polymers are extracted from biological sources, they often resemble GAGs found inside the human body. Furthermore, natural molecules possess functional side chains that allow growth promoting peptides, trophic factors and other bioactive proteins to be attached. Natural materials are often degraded safely by enzymes, whose endogenous substrates are structurally similar. For example, the utility of chitosan, a natural material derived from chitin (discussed in more detail below) has been demonstrated in studies using microcapsules containing the neurotrophic factor NT-3. Implanting neurotrophin-containing microcapsules increased regeneration of rat hippocampal neurons, supporting the safety of chitosan for *in vivo* applications [90]. Obstacles do exist for natural materials, including immunogenicity, degradation rates and mechanical properties. Common natural materials used in CNS TE applications include: alginate, chitosan, collagen, gelatin and hyaluronic acid.

In contrast to natural polymers, the degradation rates and mechanical properties of synthetic polymers are much easier to control. Encapsulating drugs or growth factors is also easier for synthetic material scaffolds. Unfortunately, these polymers are rarely used in their raw form, as they often require chemical modifications to make them biocompatible. For example, microspheres or rods comprised of poly(lactic-co-glycolic acid) (PLGA) have been successfully transplanted into the brain within minimal side effects. Glial infiltration was reported to be the same in animals that received PLGA scaffolds compared to controls [117–119]. There is some concern that PLGA degrades into acidic by-products within the brain that may exacerbate inflammation and secondary damage after brain injuries. Polycapralactone (PCL) or other polymers might be a safer alternative [120], but these are not as commonly explored as PLGA. Common synthetic materials used in CNS TE applications include poly lactic acid (PLA), poly glycolic acid (PGA), PLGA, poly ethylene glycol (PEG), PCL and others.

## Review

### Stem cell transplantation using biomaterial scaffolds

Numerous studies have been performed *in vitro* to compare the efficacy of scaffolds for neuronal differentiation and survival [97, 121, 122] however; studies are just now being published reporting the efficacy of stem cells transplanted together with a biomaterial matrix in TBI models. Each scientific team has chosen to use a different biomaterial matrix. To date, scaffolds using natural and synthetic materials constructed into rigid microparticles, rigid nanofibers and hydrogels have been tested. Moreover, in some cases the scaffolds were modified by the addition of growth factors and/or ECM molecules (Table 1).

**Table 1:** **Brain tissue engineering studies**

***Author***	***Scaffold material***	***Cell source***	***Injury model***
***Natural materials***			
[40] Tate et al.	Collagen gel + laminin/fibronectin	Mouse NPs	TBI (fluid percussion)
[42] Yu et al.	Collagen type 1 gel	E14 Rat NPs	Ischemic Stroke (MCAO)
[125] Elias et al.	Collagen scaffold	Adult hippocampal NPs	TBI (penetrative)
[126] Jin et al.	Matrigel™	Human NPs derived from ESC line	Ischemic Stroke (MCAO)
[127] Liang et al.	Hyaluronic acid	C17.2 cell line, ReNcells, and GRPs	None
[128] Wang et al.	Decellularized and lyophilized Porcine Urinary Bladder	Rat NPs	TBI (CCI)
***Synthetic materials***			
[129] Park et al.	Fibrous PLGA	C17.2	Ischemic Stroke (MCAO)
[130] Bible et al.	ppAm-PLGA microparticles + fibronectin	MHP36 cell line	Transient stroke (MCAO)
[131] Cheng TY et al.	RADA_16_-IKVAV self-assembling nanofiber	Rat neuronal progenitor cell line HCN-A94-2	Biopsy punch
***Incorporation of growth factors***			
[161] Bible et al.	PLGA + VEGF	Human cell line from 12 wk. old fetus (ReNeuron)	Ischemic Stroke (MCAO)
[170] Skop et al.	Chitosan-Heparin (genipin crosslinked) + fibronectin + FGF-2	E13.5 Rat NPs	TBI (CCI)

#### Natural biomaterials

Tate and Shear, were some of the first investigators to use stem cells for brain TE in models of TBI. They produced collagen gels that contained either fibronectin and/or laminin and showed that these scaffolds increased the survival of transplanted mouse NPs compared to NPs transplanted without the collagen matrix. The majority of cells observed weeks and months later were glia and mainly NG2+ oligodendrocyte progenitors. Tate et al. [39], obtained better long-term survival of NPs when transplanted within a supportive fibronectin and laminin matrix after TBI than transplanted directly into the brain parenchyma. Animals receiving these transplants also showed improved performance in spatial learning tasks compared to injured mice that did not receive NPs [40]. Similarly, Yu et al. [42], transplanted E14 rat NPs within a collagen type 1 hydrogel into an adult rat model of transient cerebral ischemic injury. Twenty-four hours after middle cerebral artery occlusion (MCAO) the NPs were transplanted directly into the tissue lesion with or without the scaffold. They reported that the collagen-NP scaffold promoted tissue repair better than the NPs alone. Yu et al. [42], also reported that some NPs differentiated into neurons and formed synapses, which correlated with improvements in functional recovery.

Elias et al. [123], used a similar approach repurposed for TBI. They designed a collagen scaffold to deliver adult rat hippocampal NPs in a penetrative TBI model and delivered the NPs 1 week post-injury. When examined 4 weeks later, NPs transplanted on the scaffold showed increased survival and migration compared to cells injected without the scaffold; however, neuronal engraftment was not observed as only glial and endothelial cells were observed amongst the grafted cells. They concluded that an additional growth factor or biochemical stimulus would be needed to achieve differentiated neurons *in vivo*.

Jin et al. [124], used Matrigel™ to deliver NPs to treat focal cerebral ischemia. Human NPs derived from the ESC line BG01, were transplanted within Matrigel™ scaffolds into rats recovering from MCAO by electrocoagulation 3 weeks prior. NPs transplanted within Matrigel™ decreased the infarct volume by 60%, which was superior to protection obtained when only cells or only scaffolds were injected. Moreover, cells provided with a scaffold showed greater survival and neuronal differentiation evidence by immunostaining and patch-clamp recordings. Four to 9 weeks post-transplantation improvements in sensorimotor and cognitive function were observed. While these results are promising, Matrigel™ is formed from the ECM secreted by mouse tumor cells. The specific composition is Matrigel™ is thus not suitable for clinical studies.

Another popular material being explored is hyaluronic acid, which is an abundant glycosaminoglycan in the brain. Liang et al. [125], incorporated C17.2 cells, human NP cells (ReNcells) and human glial restricted precursors into a hyaluronic acid: gelatin: polyethyleneglycol diacrylate (2:2:1) gel. C17.2 cells were transplanted into the brains of immune-competent rats whereas the ReNcells and the glial precursors were transplanted into the brains of immuno-competent mice. Survival rates for each type of precursor cell improved when encapsulated within the hydrogel.

In a very unique study, Wang et al. [126] cultured NPs in a bioreactive scaffold fabricated from porcine urinary bladders. The matrix was produced from a powder obtained after de-cellularizing and lyophilizing the bladder. De-cellularized organs (often bladders, livers and kidneys) are rich in ECM proteins like laminin, fibronectin and collagen. This powder was then rehydrated to form a hydrogel. Wang et al. [126] transplanted NPs into the brains of rats recovering from severe controlled cortical impact (CCI) injuries. This matrix supported NP proliferation and differentiation while also reducing inflammation. Furthermore, studies through 4 weeks showed improvements in motor memory and cognition that correlated with reduced tissue damage, neuron loss and white matter injury. Transplanting urinary bladder matrix alone generated the same motor and sensorimotor recovery as combined therapy; however, only the scaffold plus NPs attenuated memory and cognitive function.

#### Synthetic biomaterials

Although not as commonly employed as natural materials, synthetic materials also have been used in brain TE applications. Park et al. [127] demonstrated greater engraftment using a polymer scaffold in a stroke model. They used a fibrous poly(glycolic acid) scaffold seeded with the C17.2 cell line and showed that these cells could differentiate and reduce the extent of inflammation and glial scarring. In a more advanced study, Bible et al. [128], produced 100–200 μm PLGA microparticles that were plasma polymerized allylamine(ppAm) treated and coated with fibronectin. MHP36 NPs were transplanted on these microparticles 2 weeks after transient right MCAO. They reported that the cells integrated effectively within the host tissue and formed primitive neural tissue evidenced by Sox2, NeuN and GFAP staining. Surprisingly, Bible et al. [128] determined that transplanting MHP36 NPs into intact tissue lead to further damage.

Some groups are encapsulating NPs into self-assembling peptide hydrogels. Peptides readily self-assemble and they can form nano-fibrous networks that mimic native ECM. Moreover, like hydrogels they can be injected in soluble form and subsequently solidify to form gels *in situ*. Cheng et al. [129], linked the laminin adhesion motif isoleucine-lysine-valine-alanine-valine (IKVAV) to a self-assembling peptide AcN-RADARADARADARADA-CONH2 (RADA_16_) to form a hydrogel that possessed a mechanical stiffness similar to brain tissue. Rat NPs were encapsulated in the RADA_16_ –IKVAV hydrogel and transplanted into rats recovering from a 2 mm biopsy punch-induced neocortical injury. Histological analyses revealed that the RADA_16_ –IKVAV gel enhanced the survival of transplanted NPs (compared to NPs delivered in saline), that it reduced glial scar formation and by 6 weeks post-transplantation some of the precursors had differentiated into immature and mature neurons evidenced by βIII-tubulin, neurofilament-H, synapsin-1 and MAP2 immunostaining. Some of these cells also expressed the astrocytic marker, GFAP.

#### Incorporating growth factors

Many of the aforementioned studies reported that cell survival was often poor and neuronal differentiation difficult to achieve from transplanted neural stem and progenitor cells. Therefore, investigators have found that they need to increase the complexity of their scaffolds to incorporate survival and/or differentiation factors. Neurotrophic factors have been incorporated into biomaterial based drug delivery systems to promote nervous tissue repair. Nerve growth factor (NGF) [130–136], glial cell line-derived neurotrophic factor (GDNF) [137–142], brain derived neurotrophic factor (BDNF) [141, 143–145] and neurotrophin-3 (NT-3) [90, 135, 141, 146–148] have all been added to biomaterials to treat TBI as well as neurodegenerative disorders spinal cord injuries and peripheral nerve injuries. Growth factors such as fibroblast growth factor-2 (FGF-2), epidermal growth factor (EGF), platelet-derived growth factor (PDGF) and vascular endothelial growth factor (VEGF) also have been used for a range of CNS disorders [147–158].

In their studies, Bible et al. [159], noted that the tissue that formed wasn’t sufficiently vascularized and hypothesized that this was likely due to deficiencies in nutrients and oxygen. Therefore, in a subsequent study they incorporated 0.1% (w/v) vascular endothelial growth factor (VEGF) into the PLGA microparticles (50–100 μm) to induce angiogenesis. When transplanted 2 weeks post-MCAO [159], Bible et al., noticed significant endothelial cell infiltration and neovasularization within the tissue formed by a conditionally immortalized human NP cell line that had been transplanted on the VEGF-PLGA microparticles into the damaged hemisphere. Microglial infiltration was also observed in these conditions. Although, the VEGF promoted neurovascularizataion, neither the survival nor the differentiation of the engrafted cells was improved.

**Figure 2:**
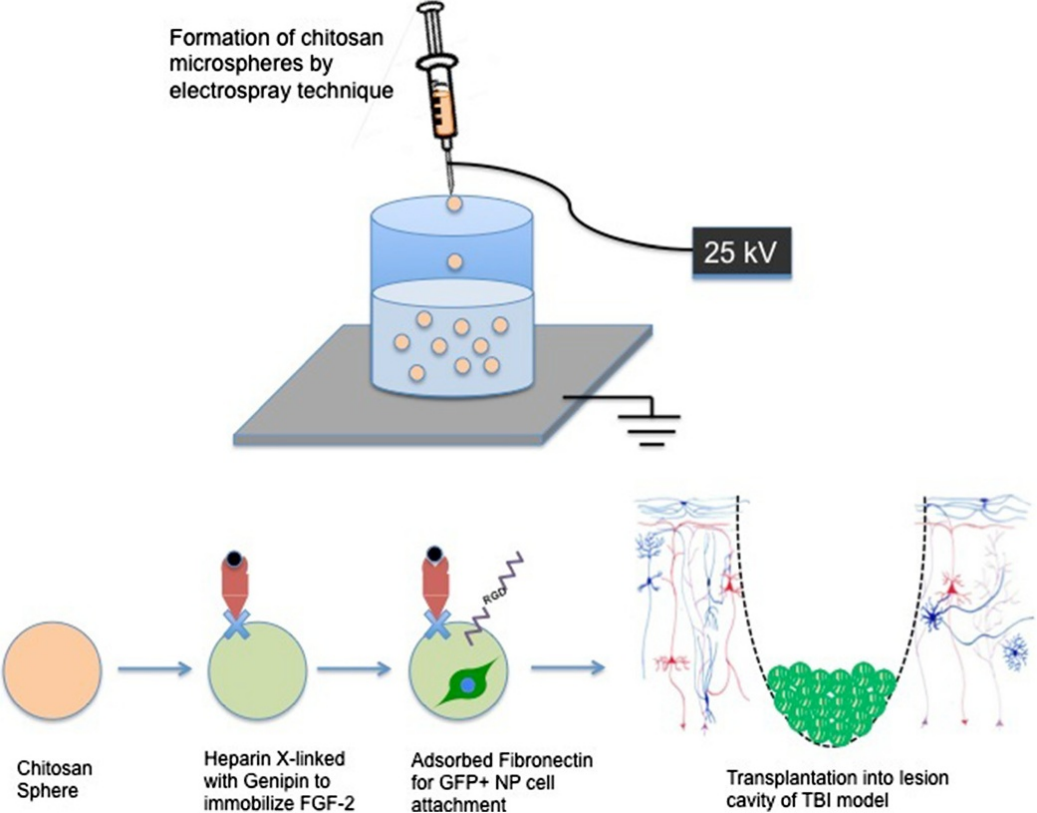
**Chitosan microsphere scaffold.** Schematic representation of the formation, modification and transplantation of chitosan microspheres for TBI repair. Chitosan is formed by electrospray technique. Heparin (red) is cross-linked to the microspheres using genipin (blue). FGF-2 (black) selectively binds to heparin. NP cells (expressing green fluorescent protein (GFP)) are attach to the scaffold by addition of fibronectin (purple). Spheres with cells are then transplanted subacutely into the lesion cavity following TBI.

For the past several years we have endeavored to produce a multifunctional microsphere scaffold optimized for transplanting NPs into the TBI brain. The scaffold that we have fabricated is produced by electrospraying a 3% chitosan solution into a coagulation bath to generate microspheres that range from 30-100 μm in diameter (mean: 64 μm). Similar gelation techniques have been used in other drug delivery methods because it is a simple process with few demerits [160–162]. Heparin, which binds FGF-2 with high affinity while retaining its biological activity, is then covalently cross-linked to the chitosan scaffolds using genipin (Figure 2). At 1 μg/mL approximately 80% of the FGF-2 binds to the scaffold. The scaffold is then further modified by coating it with fibronectin to promote cell attachment and to increase proliferation while decreasing differentiation through integrin signaling pathways. The rationale for using FGF-2, is that this growth factor is a known mitogen and survival factor for NPs and it also maintains them in a primitive state and FGF-2 also has been shown to increase the numbers of stem/progenitor cells in the SVZ following TBI [163, 164]. Interestingly, soluble FGF-2 has been reported to have a half-life of 24 hours at 32°C [165] and less than 5 hours at 37°C [166]. On the other hand, it stability increases when affixed to heparan sulfate proteoglycans [167]. Therefore, we predicted that immobilizing the FGF-2 to the scaffold would increase its biological half-life. Indeed, we have shown that fetal rat NPs plated onto a multifunctional film comprised as described above proliferated and remained multipotent for at least 3 days without providing soluble FGF-2. Moreover, they remained less mature and more highly proliferative than cells maintained on fibronectin-coated substrates in culture medium supplemented with soluble FGF-2 [168, 169].

When these multifunctional microspheres containing adherent embryonic rat radial glial cells (RGCs) that expressed green fluorescent protein were injected subacutely (7 days post injury) into the lesion cavities of adult rats that had previously sustained CCI injuries, NPs adhered to the microspheres could be observed 3 days after transplantation surrounding the microspheres and these cells were immunopositive for the primitive stem cell/progenitor markers Nestin and BLBP. Furthermore, these transplanted RGCs expressed the cell proliferation marker Ki67. At 2 weeks post-transplantation, the transplanted cells showed a reduction in stem cell/progenitor markers compared to 3 days, having acquired doublecortin (DCX), Vimentin, and Oligodendrocyte Lineage Transcription Factor 2 (Olig2), markers indicative of maturation towards neuronal, astrocytic and oligodendrocytic lineages respectively. Two weeks are still too early to expect differentiation into fully mature neurons, astrocytes, and oligodendrocytes and the continuous supply of FGF-2 from the scaffold will have delayed this process. Studies examining differentiation at 1 month post-transplantation or longer will be necessary.

We are very encouraged by these findings as they suggest that this paradigm enables balanced production of the 3 neural cell types found in the brain, whereas most groups [8, 18, 38, 39] that have transplanted primary NPs find that the majority of transplanted cells differentiate into glial cells. Furthermore, with this approach, large numbers of engrafted cells are observed that are repopulating portions of the injury and these cells are spreading across damaged neocortical regions with little clustering. This is notable, as NPs transplanted without a scaffold often remain clustered at the site of injection [89]. It is possible that the FGF-2 that is delivered on the scaffold is facilitating the migration of the NPs [40]. Several of the groups listed above also have reported greater migration of transplanted NPs when delivered using a biomaterial scaffold.

### Future directions

Significant progress has been made in neural TE, yet there are further modifications that can be made to enhance regeneration by combining stem cells and biomaterials. Four major areas of concern exist: 1) achieving a high percentage of cells that survive transplantation and persist after engraftment; 2) obtaining the correct types of differentiated cells; 3) obtaining the correct balance of the different neuronal subtypes and ensuring that they are positioned appropriately; and 4) facilitating the integration of these new neurons into existing circuits. Of these, the first 3 can be addressed by improvements in neural TE.

Progress continues to be made to address the problem of poor cell survival by modifying the biomaterial support and new innovations are being implemented that include altering the engrafted cells by introducing trophic factor genes or preconditioning the cells prior to transplantation [52]. For example, investigators have transduced NPs using lentiviral vectors encoding the neurotrophic factors BDNF, CNTF, GDNF, and NT-3 prior to implanting these cells into the injured brain [170]. Genetically engineering stem cells prior to transplantation may seem promising; however, this manipulation will create hurdles that will slow translation of this therapeutic approach into the clinic. Another approach that has been used to modify the engrafted cells is to precondition the cells. Sakata et al. [171], preconditioned NPs with interlukin-6 (IL-6) before transplanting them 6–7 hours after transient MCAO. The preconditioned NPs were protected from death and they released VEGF resulting in increased angiogenesis within the target site.

As an alternative to pre-conditioning or genetically engineering cells prior to delivery, cells can be seeded onto scaffolds that contain components necessary to induce these changes. Investigators are developing biomaterials that contain immobilized plasmids that are taken up by both the transplanted cells as well as by endogenous cells to modify their gene expression [172–174]. With this approach a variety of different gene products can be provided, and it is relatively simple to deliver multiple gene products simultaneously. Incorporating plasmids into biomaterial scaffolds has been applied towards liver [175], cartilage [176, 177], bone [178–180], skin [181], vascular/heart [182–184] and spinal cord injury [185] TE applications. Brain injuries would likely benefit from biomaterials seeded with stem cells and loaded with plasmids that would promote cell growth, neovascularization, and support donor cell survival until they can receive trophic support from their synaptic partners.

Investigators are also realizing that the developing brain is generated from a variety of different NPs; therefore, they are being more precise in matching the type of neural precursor used for engraftment. As iPSC technology becomes more refined, investigators will be able to more accurately control the type of NP that can be used for transplantation. Controlling which cell types are produced and controlling the timing of their production will require that the local environment surrounding the engrafted cells provides them with appropriate signals. Therefore, investigators will need to establish the means to deliver relevant growth and differentiation factors in a temporally defined sequence. This might require that peptides or plasmids are encapsulated for slow release from the scaffold or that they are encapsulated in a form that requires processing to become biologically active.

Another problem that needs to be overcome is to devise a strategy to regenerate a laminated brain tissue. All of the studies to date have employed basic TE strategies to enhance NP engraftment, survival and orchestrating a balanced production of neuronal and glial lineages. In order to fully repair a brain lesion, the architecture of the regenerated neural parenchyma must recapitulate the structure of the adjacent host tissue. This is especially true in the case of the neocortex, a region of the brain that is frequently damaged by trauma. The neocortex is a laminar tissue with 6 layers where the neurons located within each layer have specific neurochemical properties and they receive inputs from specific brain regions. Moreover, they send their axons to other, highly specified targets. Thus, in regenerating the neocortex, the neurons that reside in the deeper layers of the cortex (layers 5 and 6) cannot be located in more superficial regions (layers 1 through 4), and vice versa. It has been documented that NPs have the ability to sense their surroundings and reorganize to appropriately fit a cortical layer [186], though it is not likely that transplanted NPs will do the same. Therefore, new biomaterial techniques will be required to ensure the appropriate differentiation and location of NPs within the specific brain region of interest. For the neocortex, we can envision creating a multilayered scaffold, in which the different biomaterial layers govern the migration, differentiation and survival of appropriate laminar neurons. Alternatively, it might be possible to inject a biomaterial that would organize into a gradient and within this gradient, plasmids, proteins or other bioactive molecules would be organized to promote the systematic migration and differentiation of engrafted NPs [187]. Although it may be more difficult to achieve such a highly organized structure as required to repair neural circuits compared to other organ systems, utilizing TE applications to heal the injured brain remains a promising discipline for future studies.

## Conclusion

Over the past decade, TE strategies have been designed and tested for brain injury repair. Studies have shown that engraftment is improved when the stem cells are provided functionalized biomaterial scaffolds. These biomaterial scaffolds allow essential growth factors and other beneficial molecules to be delivered resulting in improved NP survival and repair. Even though natural materials have been more commonly evaluated, there is little evidence that they are superior to synthetic materials. While great progress has been achieved, additional research is necessary to determine which material(s), growth factors and/or pharmaceutical agents produce the best outcome and to determine how to best deliver them. Additional research also is needed to match the donor cells to the region of brain damage.

## Abbreviations

BDNF: Brain derived neurotrophic factor
CCI: Controlled cortical impact
CNS: Central nervous system
CNTF: Ciliary neurotrophic factor
ECM: Extracellular matrix
EGF: Epidermal growth factor
ESC: Embryonic stem cell
FGF-2: Fibroblast growth factor 2
GAG: Glycosaminoglycan
GDNF: Glial cell line-derived neurotrophic factor
iPSC: Induced pluripotent stem cell
MCAO: Middle cerebral artery occlusion
NDD: Neural degenerative disorders
NGF: Nerve growth factor
NP: Neural precursor
NSC: Neural stem cell
NT-3: Neurotrophin-3
PDGF: Platelet-derived growth factor
PLGA: Poly(lactic co-glycolic acid)
PNI: Peripheral nerve injury
RGC: Radial glial cell
SCI: Spinal cord injury
SVZ: Subventricular zone
TBI: Traumatic brain injury
TE: Tissue engineering
VEGF: Vascular endothelial growth factor
VZ: Ventricular zone.
